# The detrimental danger of water-pipe (Hookah) transcends the hazardous consequences of general health to the driving behavior

**DOI:** 10.1186/1479-5876-10-126

**Published:** 2012-06-18

**Authors:** Wafa Elias, Nimer Assy, Ibrahim Elias, Tomer Toledo, Mustafa Yassin, Abdalla Bowirrat

**Affiliations:** 1 The Ran Naor Road Safety Research Center, Technion, Haifa, Israel; 2 Ziv Medical Center, Safed, Israel; 3 Civil and Environmental Engineering, Technion, Haifa, Israel; 4 Rabin Medical Center, Campus Hasharon, Israel; 5 EMMS Nazareth-The Nazareth Hospital, Nazareth, Zip code: 16100, Israel

**Keywords:** WPS, Driving behavior, Road crashes, Carbon monoxide

## Abstract

**Objective:**

To determine whether the consumption of tobacco used in Water-Pipe by drivers increases the risk of a motor vehicle collision as a consequence of hypoxia.

**Design:**

Analytical case–control study.

**Data sources:**

Seventy exclusive Water-Pipe smokers (Experimental Group - EG) - mean age ± SD: 29.47 ± 10.45 years; mean number of weekly WPS, (6.9 ± 3.7); mean duration of WPS (WPS) is (7.5 ± 2.1 years) - and thirty non-smoker (Control Group – CG; mean age ± SD: 36.33 ± 13.92 years) were recruited during 2011 from two Arab villages located in the Galilee, northern Israel.

**Methods:**

We performed a case–control study exclusively among Water-Pipe smokers with an appropriate non smokers control group. Demographic questionnaire, Pulse Oxymeter for blood oxygenation measure and a driver simulator for measuring various participants driving behaviors were utilized. Statistical analysis for analyzing the different variables, Pearson’s *x*^2^ analysis for the comparison of categorical variables, continuous variable is compared using Student’s *t*-test and for testing the correlation between the different variables and bivariate correlation analysis were applied.

**Results:**

In the (EG) following WPS, we observed increase in the pulse rate - from 80 to 95 (t = 11.84, p < 0.05) and decrease in saturation level from 97.9 to 97.32, the decrease is statistically significant (t = 3.01, p < 0.05) versus no change in (CG). An increased number of accidents among EG (OR is 1.333 with CI of 1.008–1.776), while in CG, an insignificantly decrease (t = 3.08, p < 0.05). In EG an increase in centerline crossings (OR is 1.306 with CI of 1.016–1.679), also the total time not being within the lane was increased and the estimated (OR: 1.329; CI: 1.025–1.722). WPS increases the number of accidents by 33% and Hypoxia can cause driving behavioral turbulences.

**Conclusion:**

The results show that WPS has a significant impact on driving behavior and on the risk of being involved in road accidents and causing driving to become riskier and less careful and stable. To the best of our knowledge, this is the first time such relationships have been tested. After WPS the total number of traffic accidents and driving violations increase. The results show a significant increase in the pulse rate immediately after WPS with a decrease in the saturation rate (the level of blood oxygenation); these changes continue half an hour after WPS.

## Background

**Figure 1:**
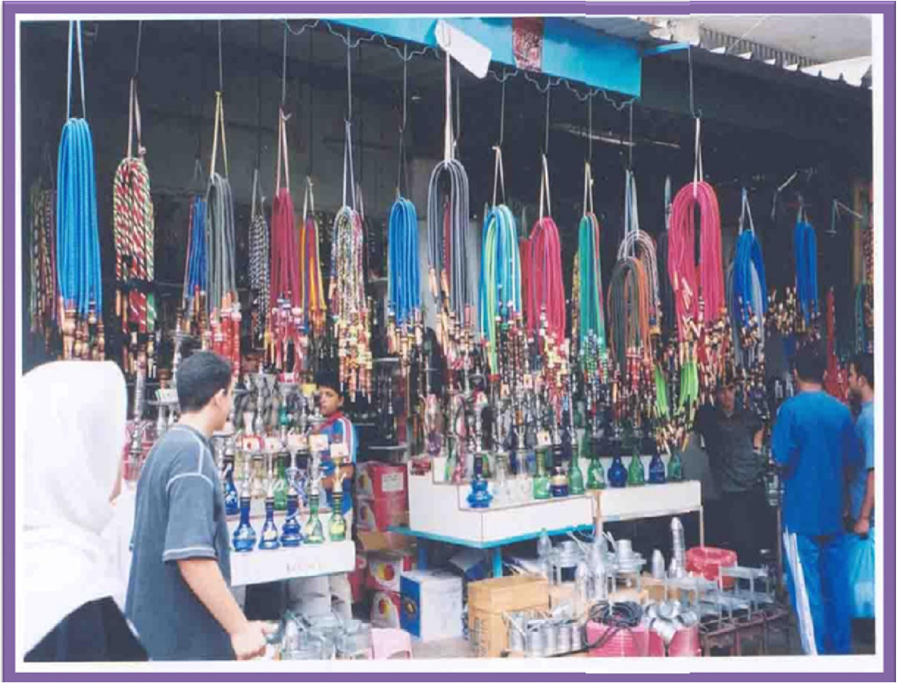
Water-Pipe store in an Arab village in northern Israel.

Water-Pipe is a device for smoking, which operates by water filtration and indirect heat of tobacco. Evidently, WPS is a major public health challenge and its use is growing in popularity but despite its highly hazardous toxic behavior has spread globally to include the African and Asian continents, Australia, Europe, and North America [1-3]. Therefore, this dangerous phenomenon no more monopoly or confined to the Eastern Mediterranean regions as reported before [2] (Figure 1).

It has been estimated that more than hundred million people globally smoke Water-Pipe daily, [4] and the global tobacco epidemic may kill 10 million people annually in the next 20–30 years, with 70% of these deaths occurring in developing countries. The composition of the tobacco used in Water-Pipe is variable and not well standardized. Studies that have examined Water-Pipe smokers and the aerosol of Water-Pipe smoke have reported high concentrations of CO, nicotine, “tar,” and heavy metals. These concentrations were as high as or higher than those among cigarette smokers. A study of CO in Water-Pipe and cigarette smoke found CO concentrations of 0.34% to 1.40% for Water-Pipe smoke and 0.41% for cigarette smoke [5]. Other studies reported elevated CO levels among Water-Pipe smokers, and the level of carboxyhemoglobin concentrations were higher among Water-Pipe smokers (10.1%) than among cigarette smokers (6.5%) or nonsmokers (1.6%), and a linear relationship was found between smoking intensity and carboxyhemoglobin concentration [5-7].

The nicotine content of Water-Pipe tobacco has been reported to be 2% to 4%, in comparison with 1% to 3% for cigarettes [8]. Other study revealed that, relative to a single cigarette, a single Water-Pipe session exposes the smoker to 1.7 times the nicotine, 3–9 times the CO, and 56-fold greater inhaled smoke volume [9-11].

As consequences of these finding, it is clear that WPS is an efficient means of delivering toxicants to the smoker. For example, after a single 45-minute WPS session, the mean plasma concentration of nicotine rose from 1.11 to 60.31 ng/mL, and cotinine rose from 0.79 to 51.95 ng/mL. Saliva nicotine concentration rose from 1.05 to 624.74 ng/mL, and cotinine rose from 0.79 to 283.49 ng/mL. The mean amounts of nicotine and cotinine excreted in a 24-hour urine sample after smoking were 73.59 μg and 249 μg, respectively [12]. According to another report, urinary cotinine concentrations were similar for Water-Pipe smokers (median of 2 pipes per day) and for cigarette smokers (median of 30 cigarettes per day) [13]. An analysis of mainstream smoke aerosol found that Water-Pipe smoke contains significant amounts of nicotine, “tar,” and heavy metals [14]. Indeed, WPS harms almost all organs in the body, causing disease, reducing quality of life and life expectancy. The emerging health risk behavior data regarding the adverse health consequences of WPS point to hazard that are similar or higher to those associated with cigarette smoking: malignancy, impaired pulmonary function, low birth weight, cardiovascular diseases, chromosomal aberrations, brain disorders and the frequent addition of alcohol or psychoactive drugs to the tobacco [10,11,15-19].

A primary behavioral pathology in drug addiction is the overpowering motivational strength and decreased ability to control the desire to obtain drugs. Addiction to tobacco smoking is influenced by a myriad of social and contextual factors, as well as the pharmacology of tobacco. Although smoking addiction has been blamed on the social influences of familial smoking and peers, current thinking is that there is also a biologic basis for these behaviors [20-22]. There is a high correlation between smoking behavior and symptoms of depression, inattention and hyperactivity in adolescents and adults [21,22]. These symptoms are often intensified during nicotine deprivation [20,23,24]. While dopamine is critical for acute reward and initiation of addiction, end-stage addiction results primarily from cellular adaptations in anterior cingulate and orbitofrontal glutamatergic projections to the nucleus accumbens. Pathophysiological plasticity in excitatory transmission reduces the capacity of the prefrontal cortex to initiate behaviors in response to biological rewards and to provide executive control over drug seeking. Simultaneously, the prefrontal cortex is hyper-responsive to stimuli predicting drug availability, resulting in supra-physiological glutamatergic drive in the nucleus accumbens, where excitatory synapses have a reduced capacity to regulate neurotransmission. In fact, cellular adaptations in prefrontal glutamatergic innervations of the accumbens promote the compulsive character of drug seeking in addicts by decreasing the value of natural rewards, diminishing cognitive control (choice), and enhancing glutamatergic drive in response to drug-associated stimuli [25]. In addition to dopaminergic effects, nicotine and well as cocaine both stimulate release of hypothalamic-anterior pituitary-gonadal and-adrenal hormones. Preclinical studies suggest that these rapid hormonal changes may contribute to the abuse-related effects of these drugs [26,27]. An improved understanding of the complex neurobiology underlying nicotine addiction is important for achieving this goal [27]. Given its high nicotine content, Water-Pipe would be expected to have a great addictive potential [16,28,29]. However, nicotine increase extracellular dopamine levels by different mechanisms. The abuse-related effects of nicotine are mediated, in part, by stimulating nicotine acetylcholine receptors (nAChRs), on the cell bodies of mesolimbic dopamine neurons in the nucleus accumbens, [30-32] and by binding to nAChRs in the ventral tegmental area, leading to stimulation of the mesolimbic dopamine system [33].

Indeed, the primary molecular target of nicotine is nAChRs, which are members of the ligand-gated ion channel super-family that includes also gamma-amino-butiric-acid, glycine, and 5-hydroxytryptamine receptors [34].

Nicotine can both activate and desensitize neuronal nAChRs, which are widely expressed in the mammalian central nervous system that mediates the physiological effects of the neurotransmitter acetylcholine (ACh) [35,36].

Functional nAChRs result from the association of five subunits each contributing to the pore lining. The major neuronal nAChRs are heterologous pentamers of (α4β2) subunits (brain), or (α3β4) subunits (autonomic ganglia). Another class of neuronal receptors that are found both in the central and peripheral nervous system is the homomeric (α7) receptor. The muscle receptor subtypes comprise of (αβδ) (embryonal) or alphabetadeltaepsilon (adult) subunits [37].

nAChRs are expressed by the first trimester in human brain and exhibit a complex pattern of developmental expression that is both region-specific and temporally regulated. In many brain areas there is a transient appearance of nAChRs during critical phases of development. Such findings suggest that acetylcholine, acting through nAChRs, may have an important functional role in modulating brain development, particularly during critical periods when brain maturation is most sensitive to perturbation. The great magnitude role of acetylcholine, acting via nAChRs, may have also vital and essential impact on the neurobiological mechanisms underlying different behavioral throughout the brain. In fact, Brody et al., reported that smoking a regular cigarette (1.2–1.4 mg nicotine) resulted in 88% occupancy of brain α_4_β_2_ nicotinic acetylcholine receptors (nAChRs) [38-40].

Indeed, the neurobiological and neurocognition mechanisms underlying the actions of nicotine are complex, involving not only the direct action of nicotine at receptors for acetylcholine but also changes in the release of other neurotransmitters, such as dopamine and glutamate [32].

Compared to the substantial volume of research on the general health consequences associated with chronic tobacco consumption, dearth research has been specifically devoted to the investigation of its effects on human neurobiology and neurocognition. Chronic tobacco consumption appears to be associated with deficiencies in executive functions, cognitive flexibility, and general intellectual abilities, to abnormal decline in reasoning, influence behavioral and mood, learning and/or memory processing speed, and working memory [41-44]. Actually, chronic smoking is related to global brain atrophy and to structural and biochemical abnormalities in anterior frontal regions, subcortical nuclei and commissural white matter. Chronic smoking may also be associated with an increased risk for various forms of neurodegenerative diseases [45]. CO is a cellular poison. It binds to hemoglobin 200–300 times more tightly than oxygen, forming COHb. As such, it inhibits the release of oxygen from hemoglobin to peripheral tissues, causing tissue hypoxia. The half life of COHb is 4 to 5 h in a person breathing room air and changes to 60 min in the presence of 100% oxygen at sea level [46]. Recent studies showed that WPS increases the individual one – CO in blood at least 5 times, compared to that from smoking a few cigarettes, and they claimed that this toxic substance can cause brain damage and loss of consciousness [47]. It is known that WPS produces more smoke than cigarette smoking. It has been estimated that smoke exposure could be as much as 100–200 cigarettes per session [48]. when the user inhales, smoke passes through the water and hose and into the lungs. Smoke inhalation can be substantial: a single Water-Pipe use episode can last 30–60 min and can involve more than one hundred inhalations, each of approximately 500 ml in volume [14,49]. Thus, while smoking a single cigarette might produce a total of approximately 500–600 ml of smoke, a single Water-Pipe use episode might produce about 50,000 ml of smoke [50]. The influence of hypoxia on physiological, behavioral, and psychological aspects of human beings has been known for decades. Hypoxia affects motor function such as abnormal motor function [51], reduced speed and precision in finger tapping. [52,53]

Also, the effects of hypoxia on cognitive functions are a typical performance decrement, difficulty in concentrating and faulty judgments [53].

For example many studies show that hypoxia prolongs the reaction time and increasing in error rates, [54] has a negative impact on cognitive abilities such as motor behavior, coordination, audition, vision and vigilance. [55-58]

In sum based on the literature, and after reviewing the impact of WPS throughout the body, we realized that Water-Pipe smokers testify to a more powerful negative effect compared to that of cigarette smoke which leads to vertigo from the very first puff. It is possible that WPS leads to stronger and deeper hypoxia which is conducive, among other things, inadequate driving, and cognitive, affective, addictive and behavioral effects changes. These changes may constitute an unconstructive influence on driving behavior and to increase risk of becoming involved in road crashes. However, according to the information available, there are no studies that have tried to explain the effect of WPS on driving and on the risk to becoming involved in road crashes. Recently a similar research related to this issue discussed but this time, the impact of other psychoactive substance (Cannabis) on driving behavior. The study demonstrated that cannabis consumption nearly doubles the risk of a collision resulting in serious injury or death [59]. The main goal of our research is to determine whether the consumption of tobacco by using Water-Pipe device by experimental group increases the risk of a motor vehicle crashes and to carry out a test of the effects of WPS on the concentration of oxygen and CO in the blood and the impact that this may have on driving behavior and the risk of becoming involved in motor vehicle collision.

## Methods

**Figure 2:**
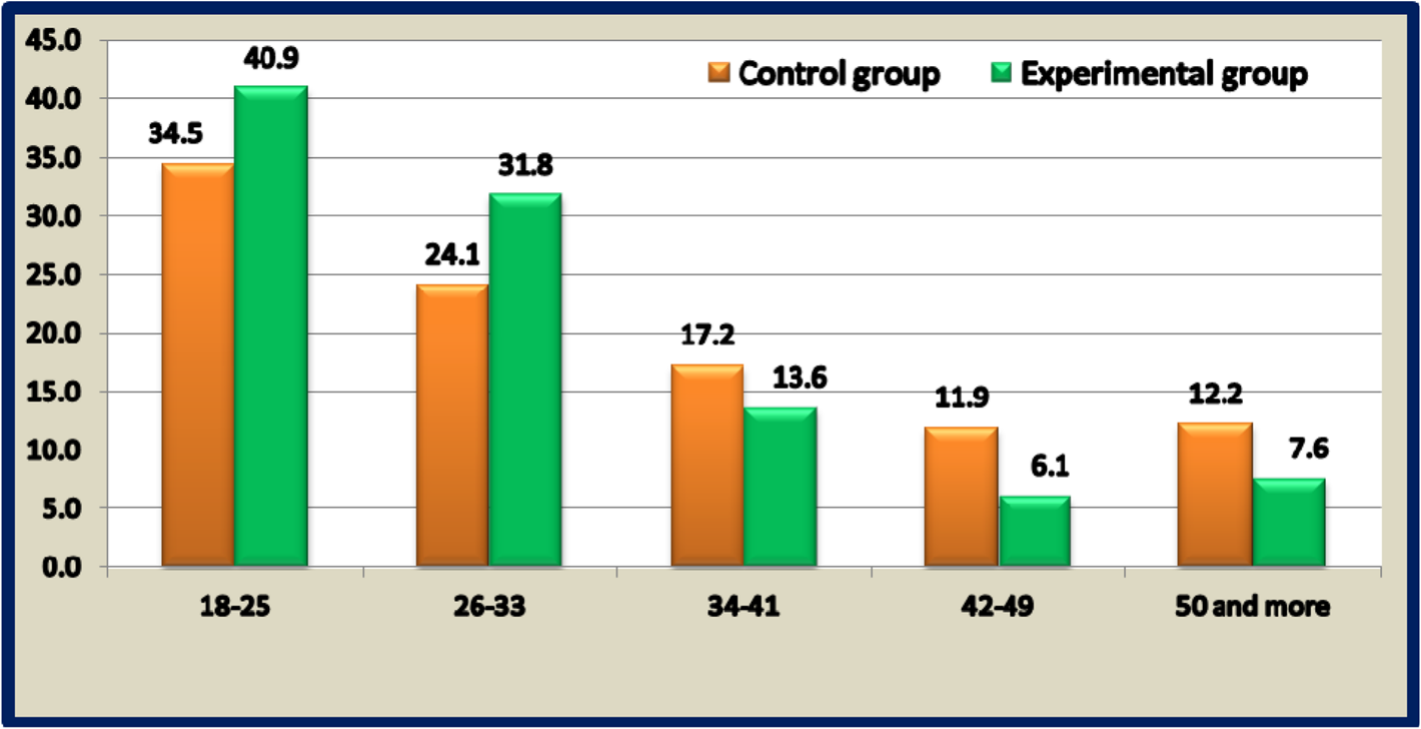
Describes and compares the age distribution for the experiment group and the control group.

A case–control study among Water-Pipe smokers with an appropriate non smokers control group was recruited. Seventy exclusive Water-Pipe smokers (Experimental Group - EG) - Mean age ± SD, 29.47 ± 10.4 years; mean number of weekly WPS, (6.9 ± 3.7); mean duration of WPS (7.5 ± 2.1 years) - and 30 non-smoker (Control Group – CG; mean age ± SD: 36.33 ± 13.92 years) were recruited from two Arab villages in the Galilee, Israel (Figure 2).

Demographic questionnaire including [Marital status, year’s average of driving, no cigarettes smoking, educational level, income, and work status, average number of cars in the household and availability of car for use (Table 1)] and Pulse Oxymeter for blood oxygenation measure in addition to a driver simulator for measuring various participants driving behaviors were utilized. Statistical analysis for analyzing the different variables, Pearson’s *x*2 analysis for the comparison of categorical variables, continuous variable is compared using Student’s *t*-test and for testing the correlation between the different variables and bivariate correlation analysis were applied.

**Table 1:** Demographic characteristics for experimental and control group

**Variable**	**Unit**	**Total sample**	**Experimental Group**	**Control group**
**Average age**	Year	31.51	29.47	36.33
**S.D.**		10.31	10.45	13.92
**Marital status**				
Married	%	45.7	44.3	50.0
Unmarried	%	52.1	52.8	50.0
Widowed	%	1.1	1.4	0
Divorced	%	1.1	1.4	0
Years of driving* (Average)	Year	11.15	9.7	14.46
**S.D.**		10.31	9.11	12.24
**No cigarettes smoking**	%	82%	81%	85%
Education level*				
0–9	%	9.0	5.71	16.66
10–12	%	60.0	62.9	43.33
Professional Diploma	%	7.0	11.43	10.00
16+	%	24.0	20.00	30.0
Income*				
Under Average	%	52.00	48.6	60.00
About Average	%	16.0	21.4	3.4
Above Average	%	20.0	18.6	23.3
No answer	%	12.0	11.4	13.3
**Work status**				
Salaried employee	%	57.0	61.4	46.7
Self-employed	%	13.0	12.8	13.3
Unemployed	%	6.0	2.9	13.3
Pensioner	%	1.0	0	3.3
Housewife	%	6.0	4.3	10
Student	%	17.0	18.6	13.4
**Average number of cars in the household**	Cars	1.85	1.83	1.87
**S.D.**		1.24	1.23	1.19
**Household size (average)**	Persons	5.25	5.4	5.07
**S.D.**		2.00	3.53	1.89
**Availability of car for your use**				
Yes	%	68.1	68.6	70.0
No	%	28.7	31.4	20.0
Sometimes	%	3.2	0.0	10.0

Here is a sample which included both the experimental and control groups. It consisted of 100 participants, whose ages ranged from 19 to 60 years (mean = 31.51; S.D = 10.31). 45.7% were married. Data analysis shows that the percentage of participants with a graduate degree (B.A., Master’s, Ph.D., or equivalent) was 24%. Most striking is that the income of 52% of the participants was below average (while the average is 8,300 Shekels per month), and 57% were salaried employees. Most of the participants (72.1%) found work outside the town; the average number of cars in the household was 1.85 (s.d = 1.24), and not surprisingly, 68.1% of the participants had a car for their use. All the participants possessed a driving license. Table 1. showed no statistical significant effect between EG and CG, for the following variables: years of driving, education and income. P-value (p = 0.284; p = 0.690 and p = 0.503 respectively).

The methodology deals with the problem with an overall approach by employing a number of methods:

 1. Testing the level of blood oxygenation using a special Pulse Oxymeter. The pulse and the level of blood oxygenation for the participants were measured three times: prior to WPS, immediately after the 30 min of WPS and 30 min subsequent to WPS.

 2. Participants completed a questionnaire including questions regarding various demographic and socio economic characteristics of the participant in the experiment such as age, gender, marital status, education, employment, income, years of smoking experience and years of driving.

 3. A driving simulator enabled the measurement of different participants’ driving behavior.

 4. In order to analyze the relationship between the different variables, descriptive statistics were employed. For a comparison between two groups, Pearson’s *x*2 analysis is used for the comparison of categorical variables, while continuous variable is compared using Student’s *t*-test. For testing the correlation between the different variables, bivariate correlation analysis was applied.

Since this study attempts to assess the effect of WPS on driving behavior, it is of great importance to establish active control for confounding variables that cannot be isolated from the main factors of interest.

 5. The importance of the control group is to account for these confounding variables, representing various differences between the participants such as in socioeconomic and demographic characteristics, years of driving experience, and years of WPS. In addition, since the experiment includes three driving scenario changes in driving behavior, perhaps as a consequence of the learning process generated by driving simulator, a control group having similar characteristics was chosen for controlling to the confounding factors.

 6. In order to estimate the effects of WPS on driving behavior, the standard epidemiological analysis of odds ratio was applied to obtain confidence intervals. The odds ratio is a way of comparing whether the probability of a certain event is the same for two groups. The odds ratio in this case is the odds of the incidents (crashes, violations) occurring in the experimental group, divided by the odds of the incidents occurring in the control group.

Equation 1 shows the typical calculation of the odds ratio

(1) $$\left(3\right)Odds\;ratio=NA_{\mathrm{In}}/NB_{\mathrm{In}}/NA_{\mathrm{Nin}}/NB_{\mathrm{Nin}}$$

Where *NA*_***In****,*_is the number of incidents in the experimental group after WPS.

NBIn, is the number of incidents in the experimental group before WPS.; NANin, is the number of incidents in the control group after WPS.; NBNin, is the number of incidents in the control group before the treatment (WPS)..

## The experiment

At the first stage, it was important to determine rules and criteria for selecting the participants.

Criteria for selecting the study participants

 1. Women and men aged 18–60 years.

 2. People who smoke a Water-Pipe (Experiment Group) and people who do not smoke a Water-Pipe (Control Group). Both groups are relatively similar (age, gender, driving experience, education level).

 3. People who sign the agreement form.

People who could not participate in this experiment

 1. People suffering from Asthma, COPD and are allergic to smoking.

 2. People with anemia.

 3. People having cardiac disease.

 4. Sufferers from cirrhosis of the liver.

 5. People with chronic renal failure.

 6. People with malignancies.

 7. Pregnant and breast feeding women.

The second stage was to prepare the driving scenarios. Three main scenarios were prepared for driving and a short scenario for the purpose of training drivers on the driving simulator. Every scenario included approximately 10 events. All the participants first drove the first scenario before smoking.

 1. The first scenario for the purpose of training was 5 km in length and included sections on inter-city and intra-city roads.

 2. The second scenario was for the purpose of driving before WPS. The length of the scenario was 10 km and included sections on inter-city and intra-city roads. The scenario additionally included a number of events (around ten) which could show changes in concentration and reaction time of drivers such as traffic lights, cars coming from a side road, pedestrians crossing the road, dogs crossing the road, cars entering the road in reverse, amounts of dirt, etc.

 3. The third scenario was for the purpose of driving immediately after WPS, its length being ten kilometers. This scenario also included approximately ten incidents, but their locations were changed.

 4. The last scenario was intended for driving half an hour after having smoked a Water-Pipe. Its length was 10 km and included about ten incidents.

Each participant smoked one head of tobacco. It was arranged that everyone smoked the same Water-Pipe tobacco with the same apple flavor (called “Double Apple,” popular in Israel and is imported from Egypt). In addition, it was important to use the same type of Water-Pipe, and of course, to smoke in the same type of environment. Also, before each scenario we examined the level of oxygen in the blood for each participant as well as the pulse rates. The outcome of the driving scenarios is a set of driving measures for every participant and every scenario. These measures indicate the changes in travel behavior.

## Data analysis based on the study survey

Socio-economic and demographic characteristics (Table 1)

## Results

Table 1. describes the demographic characteristics for EG and CG. It had showed no statistical significant effect between EG and CG, for the following variables: years of driving, education and income. P-value (p = 0.284; p = 0.690 and p = 0.503 respectively).

The level of blood oxygenation (saturation rate) using a special Pulse Oxymeter for the participants were measured in three scenarios: prior to WPS, immediately following smoking and 30 min subsequent to WPS (Table 2).

**Table 2:** The mean of the participants’ pulse rates in given WPS scenarios and non smoker controls

**Sample**	**Scenario**	**Variable**	**Mean**	**Std. Deviation**	**Std. Error Mean**
Experimental group	Before smoking	Pulse-1	80.23	13.93	1.677
	Immediately after smoking	Pulse-2	94.90	15.38	1.851
	Half hour after smoking	Pulse-3	87.18	14.39	2.036
	Before smoking	Saturation-1	97.90	.60	.072
	Immediately after smoking	Saturation- 2	97.32	1.55	.186
	Half an hour after smoking	Saturation −3	97.38	1.05	.148
Control group	Before exam	Pulse-1	82.50	11.25	2.055
	Immediately after experimental group exam	Pulse-2	80.90	9.64	1.761
	Half an hour after experimental group exam	Pulse-3	80.08	10.77	3.11
	Before exam	Saturation-1	97.57	.94	.171
	Immediately after experimental group exam	Saturation −2	97.63	.96	.176
	Half an hour after experimental group exam	Saturation-3	97.75	.45	.131

Table 2 Presents the mean of the pulse rate and the level of blood oxygenation (saturation rate) in the three scenarios: prior to smoking a Water-Pipe, immediately following smoking and half an hour subsequent to WPS in experimental group comparing to non smokers control group. In the experimental group, immediately following WPS, a statistically significant increase (Table 3) in the pulse rate was observed - from 80 to 95 (t = 11.84, p < 0.05), while in the control group a significant decrease in the pulse rate was observed - from 83 to 81. Other important results is that in the experimental group - even half an hour after Water-Pipe smoking, the pulse rate continues to be higher than that prior to Water-Pipe smoking, and the difference between the two scenarios is statistically significant (t = 5.54, p < 0.05). While in the control group, no significant change in the pulse rate was observed. In the experimental group immediately following WPS, the saturation level decreased from 97.9 to 97.32, and the decrease is statistically significant (t = 3.01, p < 0.05); while in the control group, the no significant change in the saturation rate was observed. Furthermore, in the experimental group, half an hour after WPS, the saturation rate continued to be higher than that prior to WPS and the difference is statistically significant (t = 3.02), while in the control group, no change in the saturation rate was observed half an hour subsequent to experimental group smoking a Water-Pipe.

In the experimental group, immediately following WPS, a statistically significant increase in the pulse rate was observed - from 80 to 95 (t = 11.84, p < 0.05), while in the control group a significant decrease in the pulse rate was observed - from 83 to 81 (Table 3). On the experimental group - even half an hour after WPS, the pulse rate continues to be higher than that prior to WPS, and the difference between the two scenarios is statistically significant (t = 5.54, p < 0.05). While in the control group, no significant change in the pulse rate was observed.

**Table 3:** Mean differences between the three scenarios

**Sample**	**Scenario pairs**	**t**	**Sig. (2-tailed)**	**Paired Differences**			
				**95% Confidence Interval of the Difference**		**Mean**	**Std. Deviation**
				**Upper**	**Lower**		
Control group	Pulse 1 – pulse-2	2.36	.025	2.99	.21	1.60	3.71
	Saturation-1- Saturation-2	-.57	.573	.17	−.31	−.07	.64
	pulse1 – pulse-3	1.97	.074	5.64	−.31	2.67	4.68
	Saturation1- Saturation-3	-.56	.586	.24	−.41	−.08	.51
Experimental group	Pulse-1–pulse-2	−11.84	.000	−12.20	−17.14	−14.67	10.29
	Saturation-1- Saturation-2	3.02	.004	.96	.20	.58	1.59
	pulse1 – pulse-3	−5.54	.000	−4.73	−10.11	−7.42	9.46
	Saturation1- Saturation-3	3.01	.004	.80	.16	.48	1.13

Table 2 Presents the mean of the pulse rate and the level of blood oxygenation (saturation rate) in the three scenarios: prior to smoking a Water-Pipe, immediately following smoking and half an hour subsequent to WPS in experimental group comparing to non smokers control group. In the experimental group, immediately following WPS, a statistically significant increase (Table 3) in the pulse rate was observed - from 80 to 95 (t = 11.84, p < 0.05), while in the control group a significant decrease in the pulse rate was observed - from 83 to 81. Other important results is that in the experimental group - even half an hour after Water-Pipe smoking, the pulse rate continues to be higher than that prior to Water-Pipe smoking, and the difference between the two scenarios is statistically significant (t = 5.54, p < 0.05). While in the control group, no significant change in the pulse rate was observed. In the experimental group immediately following WPS, the saturation level decreased from 97.9 to 97.32, and the decrease is statistically significant (t = 3.01, p < 0.05); while in the control group, the no significant change in the saturation rate was observed. Furthermore, in the experimental group, half an hour after WPS, the saturation rate continued to be higher than that prior to WPS and the difference is statistically significant (t = 3.02), while in the control group, no change in the saturation rate was observed half an hour subsequent to experimental group smoking a Water-Pipe.

By using the Oxymeter, the level of blood oxygenation was tested. In the experimental group immediately following WPS, the saturation level decreased from 97.9 to 97.32, and the decrease is statistically significant (t = 3.01, p < 0.05); while in the control group, the no significant change in the saturation rate was observed.

Furthermore, in the experimental group, half an hour after WPS, the saturation rate continued to be higher than that prior to WPS and the difference is statistically significant (t=, 3.02), while in the control group, no change in the saturation rate was observed half an hour subsequent to WPS.

Driving behavior using the average of the measures in the three main driving scenarios (prior to WPS, immediately following WPS and half an hour subsequent to WPS) were calculated (Table 4). These measures are the outcome of the driving scenarios for every participant and every scenario.

**Table 4:** Mean of the various driving measures for the experimental group and control groups (using the various driving measures as experimental group without smoking)

**Variable**	**Scenario**	**Experimental group**		**Control group**	
		**Mean**	**Std. Deviation**	**Mean**	**Std. Deviation**
**Before smoking**	**Accident (road)1**	**1.77**	**1.95**	**1.50**	**2.13**
**Immediately after smoking**	**Accident (road)2**	**1.30**	**1.73**	**.90**	**1.37**
**Half an hour after smoking**	**Accident (road)3**	**.71**	**.96**	**.50**	**.67**
**Before smoking**	**Accident (car)1**	**2.99**	**2.86**	**2.47**	**2.61**
**Immediately after smoking**	**Accident (car)2**	**3.06**	**2.32**	**1.87**	**1.59**
**Half an our after smoking**	**Accident (car)3**	**4.14**	**2.18**	**3.17**	**1.75**
**Before smoking**	**Accident (pedestrian)1**	**1.30**	**.91**	**1.30**	**.75**
**Immediately after smoking**	**Accident (pedestrian)2**	**.57**	**.67**	**.43**	**.50**
**Half an hour after smoking**	**Accident (pedestrian)3**	**.71**	**.71**	**.67**	**.78**
**Before smoking**	**Surpassing speed limit1**	**10.48**	**7.22**	**8.60**	**6.75**
**Immediately after smoking**	**Surpassing speed limit2**	**9.54**	**6.61**	**8.63**	**7.58**
**Half an hour after smoking**	**Surpassing speed limit3**	**12.80**	**8.01**	**11.17**	**6.09**
**Before smoking**	**Total number of traffic light tickets 1**	**1.21**	**1.07**	**1.27**	**.98**
**Immediately after smoking**	**Total number of traffic light tickets 2**	**1.12**	**.86**	**.70**	**.79**
**Half an hour after smoking**	**Total number of traffic light tickets 3**	**.69**	**.72**	**.50**	**.67**
**Before smoking**	**Centerline crossings1**	**7.03**	**6.53**	**5.87**	**4.73**
**Immediately after smoking**	**Centerline crossings2**	**8.97**	**7.69**	**7.93**	**7.04**
**Half an hour after smoking**	**Centerline crossings3**	**9.00**	**5.78**	**6.42**	**4.32**
**Before smoking**	**Shoulder crossing1**	**7.65**	**6.72**	**6.30**	**5.09**
**Immediately after smoking**	**Shoulder crossing2**	**5.80**	**4.92**	**5.50**	**4.39**
**Half an hour after smoking**	**Shoulder crossing3**	**4.88**	**4.40**	**4.25**	**3.91**
**Before smoking**	**Total time1**	**759.82**	**103.78**	**811.91**	**141.69**
**Immediately after smoking**	**Total time2**	**748.86**	**98.90**	**757.26**	**174.36**
**Half an hour after smoking**	**Total time3**	**715.13**	**121.20**	**766.01**	**174.94**
**Before smoking**	**Exceeding speed limit (%time)1**	**13.38**	**10.75**	**10.09**	**9.27**
**Immediately after smoking**	**Exceeding speed limit (%time)2**	**56.53**	**347.90**	**13.21**	**12.35**
**Half an hour after smoking**	**Exceeding speed limit (%time)3**	**17.64**	**11.22**	**14.61**	**10.10**
**Before smoking**	**Not keeping within lane (%time)1**	**7.22**	**6.67**	**6.38**	**6.01**
**Immediately after smoking**	**Not keeping within lane (%time)2**	**8.16**	**6.97**	**7.60**	**6.84**
**Half an hour after smoking**	**Not keeping within lane (%time)3**	**7.08**	**4.95**	**5.19**	**3.93**

Table 4 Presents the average of the measures in the three main driving scenarios (prior to WPS, immediately following WPS and half an hour subsequent to smoking a Water-Pipe). These measures are the outcome of the driving scenarios for every participant and every scenario. The measures include total number of road crashes, road crashes (self crash), car accidents, pedestrian accidents, surpassing the speed limit (this measure tested the number of times the driver exceeded the speed limit), the total number of traffic light violations, centerline crossings, road shoulder crossings and speed limit violations (%time). This measure indicates the percentage of time relative to the total driving time the driver surpasses the speed limit. The final measure was for not driving within the lane (%time) which showed the percentage of time relative to the total driving time the driver drove over the center divider and the shoulder boundary.

The measures include total number of road crashes, road crashes (self crash), car accidents, pedestrian accidents, surpassing the speed limit (this measure tested the number of times the driver exceeded the speed limit), the total number of traffic light violations, centerline crossings, road shoulder crossings and speed limit violations (%time). This measure indicates the percentage of time relative to the total driving time the driver surpasses the speed limit. The final measure was for not driving within the lane (%time) which showed the percentage of time relative to the total driving time the driver drove over the center divider and the shoulder boundary. Indeed, (Table 4) shows that the driving measures within both groups the experimental before WPS and the control “scenario-1” are relatively similar and the differences between the measures are statistically insignificant at to a (p-value of 0.05). While immediately after WPS and half an hour after smoking all the driving measures were higher within the experimental group than the control group, which meaning more crashes, more violation and more risky driving.

Tables 5 and 6 present the mean differences for the driving measures between the first scenario and the second scenarios (prior to WPS and immediately following it) and between the first and third scenarios (prior to WPS and half an hour following it), respectively, for the experimental and the control groups.

**Table 5:** Differences in driving behavior prior to WPS and immediately following it

**Before smoking- Immediately after smoking Pairs:scinario1-scinario2**		**Paired Differences**				**t**	**Sig. (2-tailed)**
		**Mean**	**Std. Deviation**	**95% Confidence Interval of the Difference**			
				**Lower**	**Upper**		
Control group	Accident(road)	.60	2.16	−.21	1.41	1.52	.14
	Accident(car)	.60	2.74	−.42	1.62	1.20	.24
	Accident(pedestrian)	.87	.97	.50	1.23	4.88	.00
	Exceeding speed limit	−.03	4.84	−1.84	1.77	−.04	.97
	Total number of traffic light tickets	.57	1.01	.19	.94	3.08	.00
	Centerline crossings	−2.07	6.67	−4.56	.42	−1.70	.10
	Shoulder crossings	.80	4.22	−.78	2.38	1.04	.31
	Total time	54.66	204.83	−21.83	131.14	1.46	.15
	Total distance	145.63	1152.29	−284.64	575.91	.69	.49
	Exceeding the speed limit (%time)	−3.12	9.60	−6.71	.47	−1.78	.09
	Not within the lane (%time)	−1.22	5.03	−3.09	.66	−1.32	.20
Experimental group	Accident(road)	.46	2.11	−.04	.97	1.82	.07
	Accident(car)	−.07	3.08	−.81	.67	−.20	.85
	Accident(pedestrian)	.74	1.05	.49	.99	5.83	.00
	Over speed limit	.94	6.06	−.51	2.40	1.29	.20
	Total number of traffic light tickets	.09	1.29	−.22	.40	.57	.57
	Centerline crossings	−1.94	6.77	−3.57	−.31	−2.38	.02
	Shoulder crossings	1.86	5.74	.48	3.23	2.69	.01
	Total time	10.96	98.15	−12.62	34.54	.93	.36
	Total distance	−104.06	621.30	−253.31	45.20	−1.39	.17
	Exceeding the speed limit (%time)	−43.15	343.53	−125.68	39.37	−1.04	.30
	Not within the lane (%time)	−.94	6.62	−2.53	.65	−1.17	.24

Tables 5 Presents the mean differences for the driving measures between the first scenario and the second scenarios (prior to Water-Pipe smoking and immediately following it) and between the first and third scenarios (prior to smoking a Water-Pipe and half an hour following it), respectively. From Table 5, one can see that there is an insignificant decrease in the number of road crashes immediately following Water-Pipe smoking in both the experimental and control groups, although the decrease in the control group is higher. In the experimental group, an insignificant increase in the number of car crashes was observed, but in contrast, the control group experienced a decrease. For both groups, a significant decrease in the number of pedestrian accidents was observed, but the decrease within the control group was greater than within the experimental group. In the latter group, there occurred a significant decrease in the total number of traffic light violations, while in the control group, a statistically significant decrease was observed (t = 3.08, p < 0.05).

**Table 6:** Differences in driving behavior before smoking and half an hour following a Water-Pipe

**Before Water-Pipe smoking -half an hour after smoking (Experimental Group) Pairs: Scinario-1-Scinario-3**		**Before Water-Pipe smoking -half an hour after smoking (Experimental Group) Pairs: Scinario-1-Scinario-3**		**Paired Differences**				**t**	**Sig. (2-tailed)**
				**Mean**	**Std. Deviation**	**95% Confidence Interval of the Difference**			
						**Lower**	**Upper**		
Control group	Accident(road)	.25	1.06	−.42	.92	.82	.429
	Accident(car)	−1.08	2.15	−2.45	.28	−1.74	.109
	Accident(pedestrian)	.75	1.36	−.11	1.61	1.91	.082
	Exceeding the speed limit	−4.00	6.97	−8.43	.43	−1.99	.072
	Total number of traffic light tickets	.50	1.45	−.42	1.42	1.20	.256
	Centerline crossings	−1.33	4.12	−3.95	1.28	−1.12	.286
	Shoulder crossings	.75	3.86	−1.71	3.21	.67	.515
	Total time	87.00	155.39	−11.73	185.73	1.94	.079
	Total distance	−117.17	130.86	−200.31	−34.02	−3.10	.010
	Exceeding the speed limit (%time)	−7.81	10.81	−14.68	−.94	−2.50	.029
	Not within the lane (%time)	−.43	4.04	−3.00	2.13	−.37	.718
Experimental group	Accident(road)	.90	1.81	.38	1.42	3.48	.001
	Accident(car)	−1.65	2.27	−2.30	−1.00	−5.10	.000
	Accident(pedestrian)	.45	.89	.19	.70	3.53	.001
	Exceeding the speed limit	−2.57	6.04	−4.31	−.84	−2.98	.005
	Total number of traffic light tickets	.44	1.13	.11	.77	2.69	.010
	Centerline crossings	−2.71	4.25	−3.93	−1.49	−4.47	.000
	Shoulder crossings	2.29	6.26	.49	4.08	2.56	.014
	Total time	43.19	116.26	9.79	76.58	2.60	.012
	Total distance	12.39	959.89	−263.32	288.10	.09	.928
	Exceeding the speed limit (%time)	−5.41	8.85	−7.95	−2.86	−4.28	.000
	Not within the lane (%time)	−.57	5.12	−2.04	.90	−.78	.437

Table 6 Shows the mean differences for the driving measures prior to, and half an hour following, WPS. There were no significant changes pertaining to all the measures within the control group. While in the experimental group, many significant changes in driving behavior were found, such as a decrease in the number of road crashes, a significant increase occurred in the number of car accidents, but a significant decrease in the number of pedestrian ones. In all these measures within the control group, the same direction of change was found, though this was not statistically significant. Within the experimental group, there was a significant increase in the number of incidents in which the driver exceeded the speed limit and a significant increase in the number of times the driver crossed the solid divider.

The two tables include the mean differences, standard deviation, T-statistics and the confidence intervals of the differences. In (Table 5), it was expected that all the participants will gain experience. The experience gained by drivers was expected to decrease the number of pedestrian crashes!

One can see that there is an insignificant decrease in the number of road crashes immediately following WPS in both the experimental and control groups, although the decrease in the control group is higher. In the experimental group, an insignificant increase in the number of car crashes was observed, but in contrast, the control group experienced a decrease. For both groups, a significant decrease in the number of pedestrian accidents was observed, but the decrease among the control group was greater than among the experimental group explained by difficulties in coordination, dizziness, low energy, fatigue and sleepiness, for the EG which are the results of hypoxia.

In the latter group, there occurred an insignificant decrease in the total number of traffic light violations, while in the control group, a statistically significant decrease was observed (t = 3.08, p < 0.05).

(Table 6) shows the mean differences for the driving measures prior to, and half an hour following WPS. There were no significant changes pertaining to all the measures within the control group.

While in the experimental group, many significant changes in driving behavior were found, such as a decrease in the number of road crashes, a significant increase occurred in the number of car accidents, but a significant decrease in the number of pedestrian ones. In all these measures within the control group, the same direction of change was found, though this was not statistically significant. Within the experimental group, there was a significant increase in the number of incidents in which the driver exceeded the speed limit and a significant increase in the number of times the driver crossed the solid divider.

It is important to note that comparing means is not sufficient in examining the significance of the changes in driving behavior, since during the driving process, the participants - both those who smoke a hookah and those who do not, generate an experience. Therefore, to provide a control for the drivers’ driving experience, the odds ratio test is used.

(Table 7) presents the odds ratio and the confidence interval. The odds ratio is a way of comparing whether the probabilities of the certain driving behavioral measures are the same for the two groups (the experimental and the control). An odds ratio of 1 implies that the event is equally likely in both groups. An odds ratio greater than one implies that the event is more likely in the first group, whereas an odds ratio less than one implies that the event is less likely in this group.

**Table 7:** Summary of the odds ratio test results

**Variable**	**Scinario-1-Scinario-2**			**Scinario1-Scinario-3**		
		**95% confidence interval**			**95% confidence interval**	
	**Odds ratio**	**Lower**	**Upper**	**Odds ratio**	**Lower**	**Upper**
Accidents	1.333**	1.008	1.776	1.28*	0.961	1.705
Accident(road)	1.226	0.713	2.108	1.319	0.662	2.627
Accident(car)	1.351	0.911	2.002	1.287	0.881	1.880
Accident(pedestrian)	1.289	0.634	2.621	1.195	0.607	2.351
Exceeding the speed limit	0.907	.741	1.109	0.964	0.789	1.178
Total number of traffic light tickets	1.653	0.906	3.016	1.502	0.734	3.075
Centerline crossings	0.944	0.752	1.185	1.306**	1.016	1.679
Shoulder crossings	0.867	0.678	1.110	1.001	0.758	1.322
Exceeding the speed limit (%time)	0.850	0.715	1.011	0.996	0.832	1.192
Not being within the lane (%time)	0.949	0.757	1.190	1.329**	1.025	1.722

**. Significant at the 0.05 level (2-tailed).

*. Significant at the 0.1 level (2-tailed).

Table 7 Presents the odds ratio and the confidence interval. The odds ratio is a way of comparing whether the probabilities of the certain driving behavioral measures are the same for the two groups (the experimental and the control). An odds ratio of 1 implies that the event is equally likely in both groups. An odds ratio greater than one implies that the event is more likely in the first group, whereas an odds ratio less than one implies that the event is less likely in this group. Upon comparing driving behavior before smoking a Water-Pipe and immediately after it, one can see from Table 7 that there is a significant increase in the total number of traffic accidents and the estimated OR is 1.333 with CI of 1.008–1.776 and it is statistically significant because the confidence interval did not include 1. The meaning of these results is that smoking Water-Pipe significantly increased the total number of traffic crashes by 33%. Furthermore, immediately following the smoking of a Water-Pipe, an increase in the number of the total number of traffic light tickets is found, but it is statistically significant at 0.1 and not at 0.05. The increase in measures, involvement in traffic crashes and the total number of traffic light violations indicate the risky driving of Water-Pipe smokers after having smoked a Water-Pipe.

Upon comparing driving behavior before smoking a Water-Pipe and immediately after it, one can see from (Table 7) that there is a significant increase in the total number of traffic accidents and the estimated OR is 1.333 with CI of 1.008–1.776 and it is statistically significant because the confidence interval did not include 1.

The meaning of these results is that WPS significantly increased the total number of traffic crashes by 33%. Furthermore, immediately following the WPS, an increase in the number of the total number of traffic light tickets is found, but it is statistically significant at 0.1 and not at 0.05. The increase in measures, involvement in traffic crashes and the total number of traffic light violations indicate the risky driving of Water-Pipe smokers after having smoked a Water-Pipe.

Comparing driving behavior before WPS and half an hour following it, one can see from (Table 4) that there is an increase in the total number of crashes; this is not statistically significant at 0.05 as it is borderline, while a significant increase in centerline crossings and the estimated OR is 1.306 with CI of 1.016–1.679. In addition, the percentage of the total time not being within the lane relatively to the total driving time was increased and the estimated OR is 1.329 with CI of 1.025–1.722. The meaning of these results is that half an hour after WPS the centerline crossings increased by 31% and the total time not being within the lane increased by 33%. These two measures (the centerline crossing and not being within the lane) indicate driving stability, thus post smoking drivers are less stable and their driving more dangerous. In driving behavior, these can be explained by problems with coordination, concentration, dizziness, low energy, fatigue and sleepiness, which are the results of hypoxia.

## Discussion

Consistent with previous research, [60] most water-pipe users believed erroneously that water-pipe use was neither as harmful nor as addictive as cigarette use. These perceptions of reduced risk may help explain why some individuals who do not smoke cigarettes are willing to engage in water-pipe tobacco use and, may also explain the overwhelming wave of proliferation of this phenomenon globally. International effort required to tackle the potentially hazardous health impact of this spreading jeopardy and to compact the lingering misunderstanding among the general public and especially the young group that WPS is less lethal than cigarette smoking which is no longer acceptable. The Nicotine, main stimulant psychoactive chemical ingredient in tobacco products exerts neurotoxic effects on brain. Chronic tobacco consuming appears to be associated with deficiencies in executive functions, general intellectual abilities, risk taking and sensation seeking behaviors, impairs performance of the cognitive and motor tasks necessary for safe driving and for reducing collision risk [61-64].

In addition, smoke-induced eye blurring and cough and the resultant fatigue [65,66] and even decreased vision of smokers due to deposited smoke on the automobile windshield [67].

Our results converge with recent data for road crashes that point to the increasing presence of drugs other than alcohol (especially chronic tobacco, cannabis and depressants of the central nervous system) in injured and fatally injured drivers [68-71].

Studies from Spain and also the United States have shown smokers to have a 50% higher risk of road crashes than nonsmokers [72,73].

Another study from Canada showed that 30–39 year old males who had been at-fault in crashes were 1.5 times more likely to be smokers [74].

Hence, in the context of this research, there is to shed light to the changes in the concentration of oxygen and CO in the blood following the WPS and the impact of these changes on brain function and on the risk of becoming involved in a road crash. It may be assumed that this is the first time such relationships have been tested in our area. The results show that WPS has a significant influence on driving behavior and on the risk of being involved in road crashes. Our study results also are consistent with the study hypothesis that WPS decreases the concentration of oxygen in the blood and causing general hypoxia. The results show a significant increase in the pulse rate immediately after WPS, with a decrease in the saturation rate. This result is similar to Al-Safi et al. [75] and Shafagoj & Mohammed [6] who showed that the heart rate changed from 76.40 ± 10.46 to 76.81 ± 10.19. Unsurprisingly, the effect of WPS continued for half an hour following this activity, and the results show both the pulse and saturation rates were significantly higher half an hour after WPS. The continued impact of WPS is derived from the results that have been confirmed by many studies [76] - that WPS increases the individual one – carbon dioxide in blood for at least 5 times compared to those from smoking a few cigarettes. The most important fact about one - carbon dioxide is that it has a half-life in the blood of 4 to 6 h.

Parallel to the changes in pulse and saturation rates and changes in driving behavior, it was found that immediately after WPS the total number of traffic crashes and traffic light tickets significantly increased. The increase in measures, involvement in road crashes and the total number of traffic light tickets indicate the risky driving of Water-Pipe smokers following the WPS.

This result can be explained by the stronger, deeper hypoxia caused as a result of WPS; this deeper hypoxia is conductive, among other things, to the sensation of euphoria and to inappropriate decision making and high risk talking.

The results additionally show that half an hour after WPS a significant increase in centerline crossings and the percentage of the total time not being within the lane relative to the total driving time were observed. These two measures (the centerline crossings and not being within the lane) indicate driving instability, so post WPS drivers are less stable, while their driving becomes more hazardous. Such driving behavior can be explained by problems with concentration, instability, loss of coordination, dizziness, low energy, fatigue and sleepiness which are caused as a result of the hypoxia (increase levels of CO in blood and hypoxia).

## Conclusions

 1) The results show that WPS has a significant impact on driving behavior and on the risk of being involved in road accidents and causing driving to become riskier and less careful and stable.

 2) WPS smoking increases the number of accidents by 33% and Hypoxia can cause driving behavioral turbulences.

 3) The results show a significant increase in the pulse rate immediately after WPS with a decrease in the saturation rate (the level of blood oxygenation); these changes continue half an hour after WPS.

 4) In the context of this research, there is an attempt to examine the impact of changes on the neurobiology, neurocognition, driving behaviors and on the risk of becoming involved in a road crash.

 5) These data could help inform policy-makers and interventions tackling road safety and raise public awareness of the collision risks when driving under the influence of high tobacco consumption by WPS.

## Study limitations

As this is an initial study in exploring the relationship between WPS, driving behavior and the risk of being involved in road crashes, there is need for much future work in this direction. Moreover, there is a need to broaden the sample to include more participants in order to examine the effects of additional demographic and socio-economic characteristics, such as gender, age and occupation, on WPS and driving behavior.

### Ethics approval

Ethical approval has been obtained for our manuscript.

## Competing interests

The authors declare that they have no competing interests.

## Authors’ contributions

WE – carried out the study design and participating in the recruitment of patients in addition participate in editing the manuscript and finding the manuscript. NA - Participated in the study design, and performed part of the tables and helped in performing the statistical analysis. IE- participated in the collections of data and study design, editing the paper. TT – participated in writing part of the conclusion and study design, editing the manuscript. MY- participated in the study design and editing. AB- Conceived the study, and participated in its design and coordination and wrote almost all manuscript (introduction & discussion) and helped to draft the manuscript. All coauthors have read and approved the final manuscript.
